# Reduced Serum Uric Acid and Albumin Levels in Patients with Migraine: A Cross-Sectional Study

**DOI:** 10.3390/jcm15124629

**Published:** 2026-06-15

**Authors:** Yuan-Ting Chang, Hsuan-Chu Hsu, Kuo-Cheng Lu, Yu-Chen Cheng

**Affiliations:** 1 Department of Radiology, Taipei Veterans General Hospital, Taipei 112201, Taiwan; 2 Division of Nephrology, Department of Medicine, Yangming Branch, Taipei City Hospital, Taipei 111024, Taiwan; 3 Division of Nephrology, Department of Medicine, Taipei Tzu Chi Hospital, Buddhist Tzu Chi Medical Foundation, New Taipei City 231016, Taiwan; 4 Division of Nephrology, Department of Internal Medicine, Fu Jen Catholic University Hospital, Fu Jen Catholic University, New Taipei City 243089, Taiwan; 5 School of Medicine, College of Medicine, Fu Jen Catholic University, New Taipei City 242062, Taiwan; 6 Department of Neurology, Fu Jen Catholic University Hospital, Fu Jen Catholic University, No. 69, Guizi Road, Taishan District, New Taipei City 243089, Taiwan

**Keywords:** albumin, biomarkers, cross-sectional study, migraine, oxidative stress, SATSA, total protein, twin study, uric acid

## Abstract

**Background/Objectives**: Migraine is associated with neurogenic inflammation, trigeminovascular activation, oxidative stress, and systemic metabolic changes. However, circulating antioxidant-related biomarkers in older adults with migraine remain insufficiently characterized. We examined whether self-reported migraine history was associated with serum uric acid (UA), albumin, and total protein levels in the Swedish Adoption/Twin Study of Aging (SATSA), including exploratory analyses in migraine-discordant twin pairs. **Methods**: This cross-sectional analysis used the first in-person testing wave of SATSA. Participants aged ≥50 years with complete migraine status and biomarker data were included. Serum UA was the primary outcome; albumin and total protein were secondary outcomes. Group differences were assessed using *t*-tests, Wilcoxon rank-sum tests, or chi-square tests, as appropriate. Linear regression models were adjusted for age, sex, and body mass index. Paired analyses were conducted in 13 migraine-discordant twin pairs. **Results**: Among 411 participants, 23 reported a migraine history. Participants with migraine had lower serum UA (4.39 vs. 5.15 mg/dL, *p* = 0.011), albumin (4.40 vs. 4.55 g/dL, *p* = 0.019), and total protein (7.16 vs. 7.43 g/dL, *p* = 0.008). These associations remained significant after adjustment. In discordant twin pairs, UA was lower in twins with migraine than in co-twins without migraine (4.34 vs. 4.72 mg/dL, *p* = 0.050), whereas albumin and total protein differences were not significant. **Conclusions**: Self-reported migraine history in older adults was associated with lower circulating UA, albumin, and total protein levels. These exploratory, cross-sectional findings should be interpreted as associative rather than causal and require confirmation in longitudinal studies.

## 1. Introduction

Migraine is a highly prevalent neurological disorder and a major contributor to disability worldwide. Contemporary epidemiological studies estimate a global prevalence of approximately 14–15%, with a greater burden among women and individuals in the most productive years of life [1,2]. Although migraine commonly begins earlier in life, its clinical expression may change with aging; attack frequency may decline in some individuals, whereas diagnostic uncertainty and comorbid vascular, metabolic, and neurological conditions become more prominent in older adults [3]. The recent Global Burden of Disease analysis further emphasizes the continuing public-health relevance of headache disorders and the need to better define biologically informative markers across age groups [4].

Migraine is now understood as a disorder of distributed nervous-system dysfunction involving premonitory symptoms, aura in a subset of patients, headache, and postdrome phases [5,6]. Activation and sensitization of trigeminovascular pathways, neurogenic inflammation, calcitonin gene-related peptide (CGRP) signaling, meningeal nociceptor activation, and central pain-network modulation are central to current pathophysiological models [5,6,7]. In parallel, recent literature has highlighted the contribution of autonomic nervous-system dysfunction and cranial/systemic autonomic manifestations to migraine biology, symptom burden, and potential treatment response [8,9,10,11]. These mechanisms may interact with vascular tone, inflammatory signaling, renal handling of metabolites, and systemic stress responses, but their relationship with circulating metabolic biomarkers remains incompletely understood.

Uric acid (UA), the final product of purine metabolism in humans, is biologically complex. In the extracellular compartment, UA contributes substantially to plasma antioxidant capacity through scavenging of singlet oxygen, superoxide, and other reactive species [12,13]. Experimental and translational evidence suggests that UA may exert context-dependent neuroprotective effects in models of excitotoxicity, ischemic injury, and neurodegeneration [14,15,16,17,18]. Conversely, intracellular UA accumulation and xanthine oxidase-related reactive oxygen species generation may promote oxidative stress, endothelial dysfunction, and organ injury under selected pathological conditions [19,20,21,22,23]. Thus, serum UA should not be interpreted as a simple protective or harmful marker; its biological implications depend on compartment, concentration, renal handling, oxidative context, and comorbid conditions.

Serum albumin is another major contributor to circulating antioxidant defense. Its Cys34 thiol group can buffer reactive species, and albumin also participates in transport, endothelial stabilization, and inflammatory modulation [24,25,26,27]. Lower serum albumin or total protein may therefore reflect altered nutritional, inflammatory, hepatic, renal, or systemic metabolic status rather than a migraine-specific pathway. For these reasons, associations between migraine and albumin-related markers require cautious interpretation and careful consideration of residual confounding.

Prior studies assessing UA and related antioxidant markers in migraine have yielded heterogeneous findings. Some evidence indicates lower UA levels and altered simple antioxidant blood parameters among patients with migraine [28,29,30], whereas medication-related effects, particularly topiramate-associated metabolic acidosis and changes in urate handling, may complicate interpretation [31,32]. Additional determinants of UA, including kidney function, diuretic exposure, gout, dietary purine intake, body composition, liver function, and sex-specific hormonal status, may influence observed associations [33,34,35,36,37,38,39]. These considerations are especially relevant in older cohorts, where postmenopausal status and reproductive history may contribute to metabolic variability in women.

The Swedish Adoption/Twin Study of Aging (SATSA) provides a unique opportunity to examine circulating biomarkers in older adults while also enabling exploratory within-pair analyses in twins discordant for migraine history [40]. Building on preliminary findings presented at the World Congress of Neurology 2023 [41], the present study aimed to assess whether self-reported migraine history was associated with serum UA, albumin, and total protein levels in older adults. We additionally examined migraine-discordant twin pairs as an exploratory strategy to partially account for shared genetic and early-life environmental factors. Because the study is cross-sectional and migraine status was self-reported, all findings are interpreted as associative and hypothesis-generating.

## 2. Materials and Methods

### 2.1. Data Source, Study Population, and Design

This study was a cross-sectional secondary analysis of the first in-person testing wave (IPT1) of the Swedish Adoption/Twin Study of Aging (SATSA). SATSA is a longitudinal twin study initiated in 1984 to investigate genetic and environmental influences on aging-related phenotypes [40]. The IPT1 wave was conducted between 1986 and 1988 and included structured interviews, clinical assessment, and laboratory measurements in older adults.

**Figure 1:**
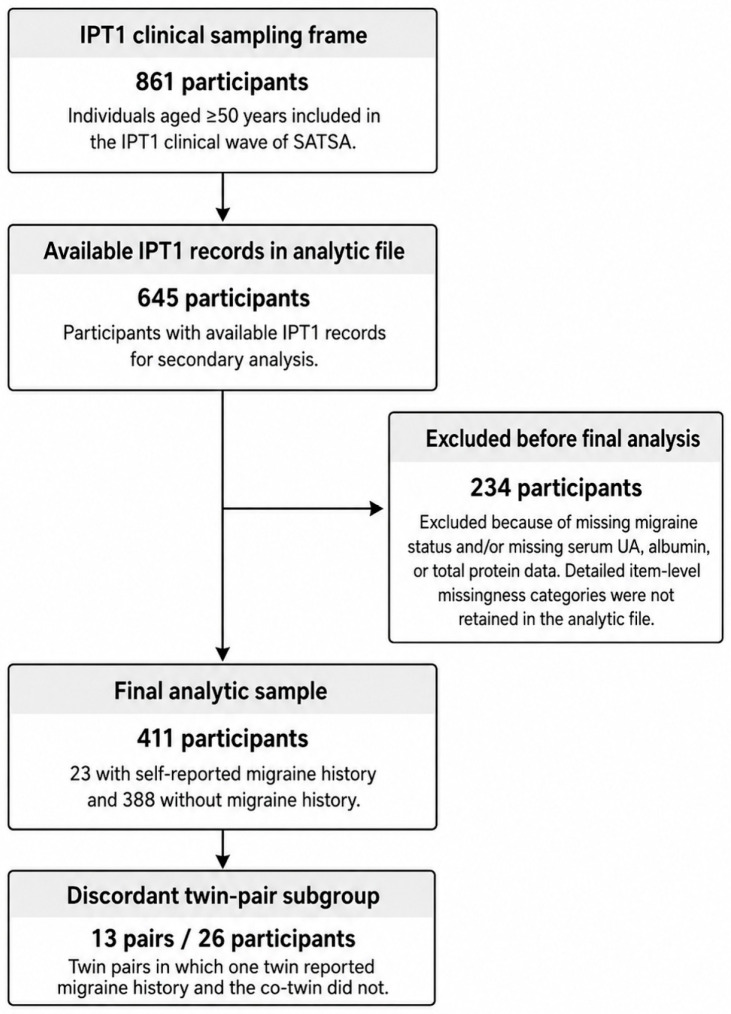
Participant flow diagram for the analytic sample and the migraine-discordant twin-pair subgroup.

To clarify participant selection (Figure 1), the IPT1 clinical sampling frame included 861 individuals aged 50 years or older. Of these, 645 participants had available IPT1 records in the analytic file used for the present study. We then restricted the sample to participants with non-missing migraine status and complete serum UA, albumin, and total protein data. A total of 234 individuals were excluded due to missing migraine or biomarker data, leaving 411 participants for the final analytic sample. Among these, 23 reported a history of migraine, and 388 did not. For the genetically informed exploratory analysis, all twin pairs in the final analytic sample were screened, and 13 twin pairs discordant for migraine history were identified and included. Baseline characteristics of included and excluded participants are compared in Supplementary Table S1.

The original SATSA protocol was approved by the Ethics Committee at Karolinska Institutet and the Swedish Data Inspection Board, and all participants provided informed consent at the time of original data collection. For this de-identified secondary analysis, the dataset was accessed and analyzed between 1 August 2019 and 31 July 2020. The Institutional Review Board of Fu Jen Catholic University approved the protocol and deemed the study exempt (IRB number: C108004). The authors had no access to information that could identify individual participants. Reporting was guided by the Strengthening the Reporting of Observational Studies in Epidemiology (STROBE) recommendations.

### 2.2. Migraine Exposure

Migraine exposure was defined using self-reported medical history obtained during structured IPT1 interviews. The SATSA dataset did not include confirmation according to the International Classification of Headache Disorders criteria, nor did it provide migraine subtype, aura status, frequency, duration, attack severity, age at onset, or migraine-specific medication data. Therefore, the exposure variable should be interpreted as a self-reported history of migraine rather than a clinically adjudicated migraine diagnosis. Prior validation work suggests that self-reported migraine can show reasonable agreement with criteria-based classification in large epidemiological cohorts [42], but misclassification remains possible and is addressed as a major limitation.

### 2.3. Biomarkers and Laboratory Measurements

Whole-blood samples were collected during the IPT1 clinical evaluation and stored at −80 °C before biochemical analysis. The primary outcome was serum UA concentration. Serum albumin and total protein were analyzed as secondary outcomes because of their roles in circulating antioxidant capacity, protein/nutritional status, and inflammatory physiology. Renal function was assessed using estimated glomerular filtration rate (eGFR), calculated using the Modification of Diet in Renal Disease equation: eGFR = 175 × Scr^−1.154^ × age^−0.203^ × 0.742 if female × 1.212 if Black. Liver function markers, including glutamic-oxaloacetic transaminase (GOT), gamma-glutamyl transferase (GGT), and total bilirubin, were also extracted.

### 2.4. Covariates and Potential Confounders

Covariates were selected based on biological plausibility and prior evidence linking them to migraine, UA metabolism, serum albumin, or systemic metabolic status. Baseline demographic and clinical variables included age, sex, BMI, educational attainment, socioeconomic status (SES), smoking history, hypertension, heart failure, myocardial infarction, diabetes, chronic liver disease, chronic kidney disease, stroke, gout, eGFR, GGT, GOT, and total bilirubin. Age was categorized as >65 versus ≤65 years, BMI as ≥25 versus <25 kg/m^2^, and smoking status as ever versus never smoking. Educational attainment and SES were coded according to the SATSA documentation [40].

Medication use, including topiramate, non-steroidal anti-inflammatory drugs, allopurinol, diuretics, antihypertensives, and hormone therapy, was not available in the public-use dataset. Dietary purine intake, alcohol intake, menopausal status, reproductive history, and direct measures of urinary UA excretion were also unavailable. These unmeasured variables were therefore not included in the regression models and are treated as sources of potential residual confounding.

### 2.5. Statistical Analysis

Descriptive statistics were used to summarize baseline characteristics. Categorical variables were compared using Pearson’s chi-square test or Fisher’s exact test when cell counts were small. Continuous variables were assessed for distributional assumptions by visual inspection of histograms and Q-Q plots and, when appropriate, by Shapiro–Wilk tests. Normally distributed continuous variables were compared using Student’s *t*-test, whereas skewed variables were compared using the Wilcoxon rank-sum test.

Associations between migraine history and serum biomarkers were evaluated using crude and multivariable linear regression models. The prespecified adjusted model included age, sex, and BMI. We considered broader adjustments for eGFR, chronic kidney disease, hypertension, diabetes, stroke, smoking, gout, SES, and liver function markers; however, because only 23 participants had migraines, extensive multivariable adjustment could increase model instability and overfitting. Therefore, these variables were summarized descriptively and incorporated into the interpretation rather than entered simultaneously into the primary regression model.

To partially account for shared genetic background and early-life environmental factors, an exploratory paired analysis was conducted in 13 twin pairs discordant for migraine history. Paired *t*-tests compared serum UA, albumin, and total protein levels within twin pairs. Given the small subgroup size, these paired analyses were considered exploratory and hypothesis-generating. Post hoc power analyses were performed based on observed effect sizes at alpha = 0.05. All tests were two-sided, and *p* < 0.05 was considered statistically significant. Analyses were conducted using R version 3.5.1 (R Foundation for Statistical Computing, Vienna, Austria) in the RStudio version 1.3.959 (RStudio, Inc., Boston, MA, USA). As sensitivity analyses to assess the robustness of findings against residual confounding, we repeated the primary comparisons after excluding participants with self-reported stroke history (Sensitivity 1, *n* = 406) and after simultaneously excluding those with self-reported stroke, diabetes mellitus, gout, kidney disease, or liver disease (Sensitivity 2, *n* = 351).

### 2.6. AI-Assisted Language Editing Statement

During manuscript revision, the authors used an AI-based language tool (ChatGPT, GPT-4o, OpenAI, San Francisco, CA, USA) only to assist with English-language editing, organization, and clarity of presentation. The tool was not used to generate new data, perform statistical analyses, select the study population, or make independent scientific conclusions. All scientific content, interpretations, references, and final wording were reviewed, verified, and approved by the authors, who take full responsibility for the manuscript.

## 3. Results

Among the 411 participants included in the final analytic sample, 23 (5.6%) reported a history of migraine, and 388 did not. The participant-flow process is shown in Figure 1. Baseline demographic and clinical characteristics are summarized in Table 1. Participants with migraine were slightly younger and more frequently female than those without migraine, but these differences were not statistically significant. BMI category, educational attainment, SES, smoking history, hypertension, diabetes, chronic kidney disease, and gout did not differ significantly between groups.

Stroke was more frequent among participants with migraine than among those without migraine (8.7% vs. 0.8%, *p* = 0.027). Liver function markers also differed between groups: participants with migraine had lower GGT levels and higher GOT levels, whereas total bilirubin did not differ significantly. Because these variables may reflect comorbid conditions or systemic physiology relevant to biomarker interpretation, they were considered in the Discussion as possible sources of residual confounding.

In crude comparisons, participants with migraine had lower serum UA levels than participants without migraine (4.39 vs. 5.15 mg/dL, *p* = 0.011). Serum albumin (4.40 vs. 4.55 g/dL, *p* = 0.019) and serum total protein (7.16 vs. 7.43 g/dL, *p* = 0.008) were also lower in participants with migraine. In regression analyses adjusted for age, sex, and BMI, migraine history remained associated with lower serum UA (estimate −0.552 mg/dL, *p* = 0.049), albumin (estimate −0.151 g/dL, *p* = 0.019), and total protein (estimate −0.264 g/dL, *p* = 0.009) (Table 2). Post hoc power analyses indicated sufficient statistical power for serum UA (97.6%) and acceptable power for total protein (87.5%) and albumin (75.9%) at alpha = 0.05.

In the exploratory analysis of 13 migraine-discordant twin pairs, twins with migraine had a lower mean serum UA than their co-twins without migraine (4.338 vs. 4.715 mg/dL, *p* = 0.050). Albumin and total protein were numerically lower among twins with migraine, but these differences were not statistically significant (albumin: 4.423 vs. 4.438 g/dL, *p* = 0.808; total protein: 7.085 vs. 7.269 g/dL, *p* = 0.199) (Table 3). Given the small number of discordant pairs, these within-pair findings should be interpreted cautiously.

In sensitivity analyses, the observed associations were consistent after excluding participants with stroke history (*n* = 406) and after excluding participants with any of the following conditions: stroke, diabetes mellitus, gout, kidney disease, or liver disease (*n* = 351). All three biomarker associations remained statistically significant across both sensitivity analyses (Table S2).

## 4. Discussion

In this cross-sectional analysis of older adults from SATSA, self-reported migraine history was associated with lower serum UA, albumin, and total protein levels. These associations remained statistically significant after adjustment for age, sex, and BMI. An exploratory within-pair analysis of migraine-discordant twins showed a similar direction for serum UA, although albumin and total protein differences were not statistically significant. Collectively, these findings suggest that migraine history may be associated with an altered circulating antioxidant-related and protein-metabolic profile in older adults, but the observational design precludes causal inference.

The lower serum UA observed among participants with migraine is consistent with prior studies reporting reduced UA or altered antioxidant blood parameters in migraine populations [28,29,30,43,44]. UA contributes to extracellular antioxidant capacity and may protect neurons under selected experimental conditions [13,17,18]. However, UA biology is context-dependent: intracellular accumulation and xanthine oxidase-related ROS generation may promote oxidative stress and vascular dysfunction [19,20,21,22,23,45]. Therefore, the present results should not be interpreted as proof of antioxidant depletion, neuroprotective loss, or a mechanistic causal pathway. A more cautious interpretation is that migraine history was associated with lower circulating UA in this older cohort.

Lower albumin and total protein levels may also be consistent with altered systemic antioxidant, inflammatory, nutritional, hepatic, or renal physiology. Albumin contributes substantially to plasma redox buffering through its free thiol group and participates in transport and inflammatory regulation [24,25,26]. Nevertheless, serum albumin is a nonspecific biomarker and can be influenced by age, comorbid disease, inflammatory burden, nutritional status, liver function, renal protein loss, and medication use [25]. The present study cannot distinguish among these possibilities.

Several unmeasured determinants of UA and albumin deserve emphasis. Medication data were unavailable, including topiramate, diuretics, allopurinol, antihypertensive agents, NSAIDs, and hormone therapy. Topiramate and diuretics may influence acid–base balance, urinary citrate, urate handling, and kidney-stone risk [31,32,33,46,47]. Kidney function and renal excretion dynamics are also central to UA homeostasis [48]. Although eGFR and chronic kidney disease were descriptively assessed, detailed urinary UA excretion data were not available. Dietary purine intake, alcohol consumption, and detailed body-composition measures were likewise unavailable.

Sex-specific and hormonal factors are particularly relevant because most participants with migraine were women and because the cohort consisted of older adults. Menopause, estrogenic signaling, postmenopausal hormone therapy, parity, miscarriage, and broader reproductive history may influence UA regulation and midlife metabolic or physiological profiles [36,37,38,39]. The SATSA dataset used here did not contain sufficient reproductive or hormonal data for adjustment. Consequently, residual confounding by menopausal status, hormone therapy, and reproductive history remains possible and should be considered when interpreting the results.

The difference in stroke prevalence between groups also requires caution. Stroke was uncommon overall but was more frequent among participants with migraine. Stroke history and vascular comorbidity may influence inflammatory status, nutritional status, renal physiology, mobility, and circulating biomarkers [49]. Because only 23 participants had migraines, fully adjusted models including multiple comorbidities would be statistically unstable. Future studies should include larger samples that permit multivariable adjustment for vascular disease, renal function, hepatic markers, smoking, gout, medication use, diet, and socioeconomic variables. Although hyperuricemia and gout have been associated with an increased risk of stroke—with recent evidence demonstrating a higher incidence of gout flares in acute stroke patients, particularly among men, potentially reflecting high-grade systemic inflammation [50]—our study was not designed to evaluate stroke risk per se. Rather, we focused on circulating antioxidant biomarkers in older adults with a self-reported migraine history, the majority of whom were women. In this context, lower serum UA levels may reflect reduced extracellular antioxidant capacity and greater oxidative stress in migraine-related pathophysiology [13,28,44]. Therefore, our finding of lower serum UA levels in patients with migraine does not necessarily contradict previous findings linking hyperuricemia or gout to stroke risk. Rather, these observations suggest that the relationship between uric acid and neurovascular disorders may be nonlinear and disease-context-dependent.

The autonomic nervous system may provide an additional conceptual link between migraine and systemic physiology. Recent reviews emphasize that migraine can involve central and peripheral autonomic pathways, cranial autonomic symptoms, CGRP-related neuroinflammation, and systemic manifestations [8,9,10,11,44,51]. These pathways could plausibly interact with vascular tone, oxidative stress, renal handling, and inflammatory signaling. However, the present dataset did not include autonomic testing, CGRP measurements, urinary biomarkers, cerebrospinal fluid biomarkers, or attack-timing information. Therefore, any autonomic or trigeminovascular explanation remains speculative.

The exploratory discordant twin-pair analysis partially addresses shared genetic and early-life environmental factors [52,53]; however, it should be regarded as strictly exploratory. The *p* = 0.050 finding for uric acid demonstrates a trend directionally consistent with the primary analysis but does not constitute strong support, given the very small number of discordant pairs (*n* = 13) and the correspondingly limited statistical power. The within-pair analysis should therefore be viewed as hypothesis-generating rather than confirmatory.

A hypothetical conceptual model is provided in Supplementary Figure S1 to summarize possible relationships among migraine, oxidative stress, autonomic/trigeminovascular activation, renal handling, and circulating biomarker differences. This model is intended only as a framework for future research and should not be interpreted as evidence of causality in the present study.

### 4.1. Limitations

This study has several important limitations. First, the cross-sectional design prevents the determination of temporality or causality. We cannot determine whether lower serum UA, albumin, and total protein preceded migraine, resulted from migraine-related physiology, or reflected shared confounding factors. Second, only 23 participants reported migraine history, limiting statistical power and preventing broad multivariable adjustment or subgroup analyses by sex, age, vascular disease, renal function, or medication exposure. Selection bias related to missing data is unlikely to have substantially biased the primary associations of interest. A formal post hoc comparison of included (*n* = 411) and excluded (*n* = 234) participants among those with available IPT1 records showed no significant differences in serum uric acid, albumin, or total protein levels (all *p* > 0.15) and comparable distributions of sex, BMI, smoking, and stroke history (Supplementary Table S1). Included participants were slightly older (66.3 vs. 64.3 years) and had higher diabetes prevalence (6.0% vs. 2.2%); however, these modest differences are unlikely to explain the observed biomarker associations, given the sensitivity analyses presented above.

Third, migraine status was self-reported and not confirmed by a clinical interview or International Classification of Headache Disorders criteria. Data on aura status, episodic versus chronic migraine, attack frequency, severity, disease duration, age at onset, and timing relative to blood sampling were unavailable. Fourth, the dataset lacked information on medications that could substantially influence UA or albumin, including topiramate, diuretics, allopurinol, antihypertensives, NSAIDs, and hormone therapy.

Fifth, several biologically important confounders were unavailable or incompletely characterized, including menopausal status, reproductive history, dietary purine intake, alcohol consumption, urinary UA excretion, inflammatory markers, and body-composition measures. Sixth, the cohort consisted of older Swedish twins; therefore, generalizability to younger patients, non-European populations, clinically confirmed migraine cohorts, or patients with chronic migraine may be limited. Finally, the twin-pair analysis was small and exploratory.

### 4.2. Future Directions

Future studies should use prospective longitudinal designs to determine whether circulating UA, albumin, and total protein predict incident migraine, migraine persistence, or migraine remission in older adults. Larger cohorts should include validated migraine phenotyping, migraine subtype, aura status, attack frequency, severity, and medication exposure. Simultaneous measurement of serum, urine, and, where clinically appropriate, cerebrospinal fluid biomarkers could help distinguish antioxidant consumption from renal excretion or redistribution mechanisms.

Future work should also incorporate hormonal and reproductive variables, including menopausal status, hormone therapy, age at menopause, parity, and miscarriage history, particularly in female participants. Integration of autonomic testing, CGRP-related biomarkers, inflammatory markers, renal handling indices, and dietary information may help clarify whether the observed associations reflect migraine biology, comorbid disease, medication effects, or broader systemic metabolic variation.

## 5. Conclusions

In this cross-sectional analysis of older adults from SATSA, self-reported migraine history was associated with lower serum UA, albumin, and total protein levels. The findings are exploratory and should be interpreted as associations rather than evidence of causal antioxidant depletion or neuroprotective loss. External validation is needed in larger, prospectively followed cohorts with clinically confirmed migraine diagnoses, detailed medication assessment, renal and urinary biomarker data, and sex-specific hormonal and reproductive profiling. Simultaneous measurement of serum, urine, and, where feasible, cerebrospinal fluid biomarkers in longitudinal designs would be essential to characterize the temporal relationship between migraine and the observed biomarker alterations.

## Figures and Tables

**Table 1:** Comparison of demographic and clinical characteristics between participants with and without migraine.

Characteristic	Participants with Migraine (*N* = 23)	Participants Without Migraine (*N* = 388)	*p*-Value
Age, years, mean (SD)	64.19 (6.18)	66.45 (8.59)	0.108
Age > 65 years, No. (%)	11 (47.8%)	226 (58.2%)	0.443
Female, No. (%)	16 (69.6%)	235 (60.6%)	0.522
BMI ≥ 25 kg/m^2^, No. (%)	9 (39.1%)	209 (54.1%)	0.240
Education attainment > O-level/vocational/folk school, No. (%)	3 (13.0%)	32 (7.4%)	0.727
Socioeconomic status, mean (SD)	0.88 (2.21)	0.12 (2.68)	0.168
Ever smoker, No. (%)	12 (52.2%)	157 (41.1%)	0.408
Hypertension, No. (%)	5 (21.7%)	68 (17.5%)	0.578
Heart failure, No. (%)	0	20 (5.2%)	0.617
Myocardial infarction, No. (%)	1 (4.3%)	11 (2.8%)	0.504
Diabetes, No. (%)	0	19 (4.9%)	0.615
Chronic liver disease, No. (%)	0	4 (1.0%)	1.000
Chronic kidney disease, No. (%)	3 (13.0%)	29 (7.5%)	0.409
Stroke, No. (%)	2 (8.7%)	3 (0.8%)	0.027
Gout, No. (%)	1 (4.3%)	2 (0.5%)	0.159
eGFR, mL/min/1.73 m^2^, mean (SD)	61.10 (10.14)	56.87 (12.24)	0.066
GGT (U/L), median (IQR)	14.0 (10.5–20.5)	18.0 (12.0–27.0)	0.037 ^a^
GOT (U/L), median (IQR)	13.0 (11.5–16.0)	10.0 (7.0–14.0)	0.001 ^a^
Total bilirubin (mg/dL), median (IQR)	0.40 (0.35–0.55)	0.50 (0.30–0.60)	0.890 ^a^
Serum uric acid (mg/dL), mean (SD)	4.39 (0.86)	5.15 (1.41)	0.011
Serum albumin (g/dL), mean (SD)	4.40 (0.26)	4.55 (0.30)	0.019
Serum total protein (g/dL), mean (SD)	7.16 (0.40)	7.43 (0.47)	0.008

^a^ *p*-value calculated using the Wilcoxon rank-sum test. Abbreviations: BMI, body mass index; eGFR, estimated glomerular filtration rate; GGT, gamma-glutamyl transferase; GOT, glutamic-oxaloacetic transaminase; IQR, interquartile range; SD, standard deviation.

**Table 2:** Linear regression analysis of the association between migraine history and serum biomarkers.

Biomarker	Crude Estimate (SE)	Crude *p*-Value	Adjusted Estimate (SE) *	Adjusted *p*-Value
Serum uric acid (mg/dL)	−0.761 (0.297)	0.011	−0.552 (0.280)	0.049
Serum albumin (g/dL)	−0.150 (0.064)	0.019	−0.151 (0.064)	0.019
Serum total protein (g/dL)	−0.268 (0.100)	0.008	−0.264 (0.100)	0.009

* Adjusted for age, sex, and BMI. Abbreviations: BMI, body mass index; SE, standard error.

**Table 3:** Comparison of serum biomarkers in twin pairs discordant for migraine history.

Biomarker	Twins with Migraine (*N* = 13)	Co-Twins Without Migraine (*N* = 13)	*p*-Value
Serum uric acid (mg/dL), mean (SD)	4.338 (0.683)	4.715 (0.709)	0.050
Serum albumin (g/dL), mean (SD)	4.423 (0.283)	4.438 (0.253)	0.808
Serum total protein (g/dL), mean (SD)	7.085 (0.391)	7.269 (0.544)	0.199

Abbreviations: SD, standard deviation. The twin-pair analysis was exploratory because of the limited number of discordant pairs.

## Data Availability

The SATSA cohort data for in-person testing waves are available through the Inter-university Consortium for Political and Social Research/NACDA Program on Aging: https://www.icpsr.umich.edu/icpsrweb/ICPSR/studies/3843 (accessed on 8 June 2026). Additional study information is available through the Maelstrom Research platform: https://www.maelstrom-research.org/mica/individual-study/satsa (accessed on 8 June 2026).

## Supplementary Materials

The following supporting information can be downloaded at: https://www.mdpi.com/article/10.3390/jcm15124629/s1. Supplementary Figure S1: Proposed hypothetical pathways between migraine and lower serum uric acid and albumin; Table S1: Baseline characteristics of included versus excluded participants among those with available IPT1 records (*n* = 645); Table S2: Sensitivity analyses: serum uric acid, albumin, and total protein by migraine status.
