# Supplementary material for: Reduced Serum Uric Acid and Albumin Levels in Patients with Migraine: A Cross-Sectional Study
Source: J Clin Med. 2026 Jun 15;15(12):4629. doi: 10.3390/jcm15124629 (PMC13302064; doi:10.3390/jcm15124629)
Supplement: Supplementary file 1 [file jcm-15-04629-s001.zip › jcm-4283058-supplementary.pdf]

Supplementary Figure S1. Proposed hypothetical pathways between migraine and lower serum uric acid and albumin. A hypothetical conceptual model linking potential autonomic and trigeminovascular pathways to systemic antioxidant variations. Genetic variations associated with vascular and smooth muscle tissues may contribute to individual susceptibility to migraine. Hypothetically, during migraine episodes, the release of calcitonin gene-related peptide (CGRP), substance P, and other neuropeptides from trigeminal ganglion cells might interact with pro-inflammatory factors and meningeal vasodilation. It is hypothesized that chronic neuro-inflammatory signaling and potential systemic endothelial variations could collectively influence peripheral antioxidant consumption, potentially manifesting as lower serum albumin, total protein, and uric acid levels.

This conceptual model is strictly speculative and hypothesis-generating; it is intended solely to guide future longitudinal investigations and should not be interpreted as evidence of biological determinism or causal mechanisms within the present cross-sectional study.

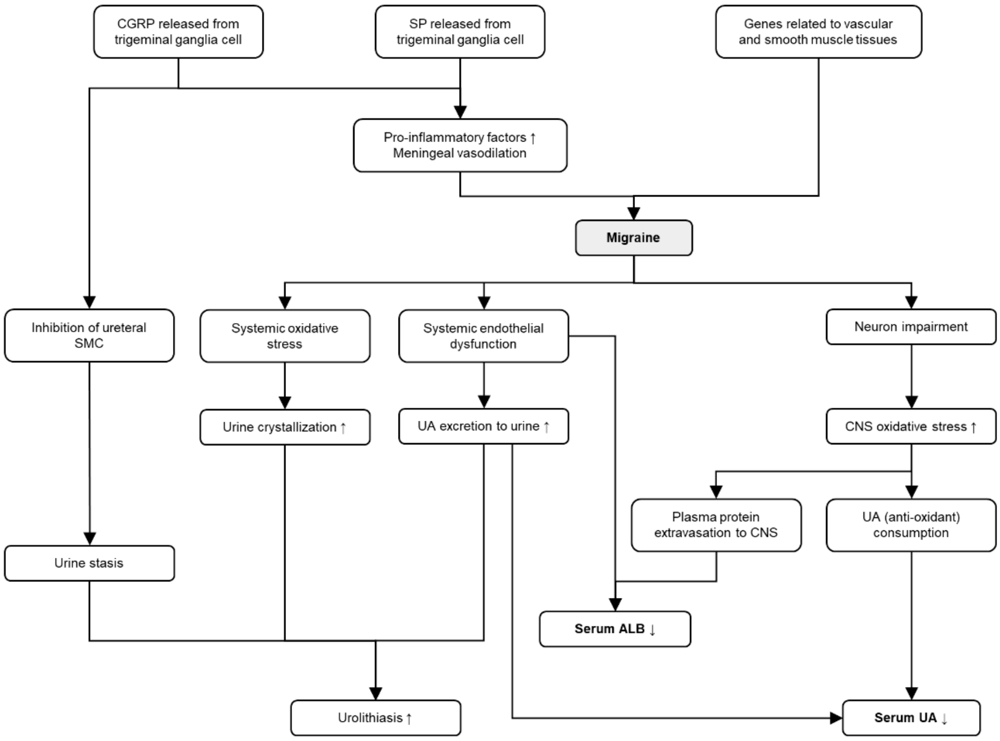

Table S1. Baseline characteristics of included versus excluded participants among those with available IPT1 records (n = 645).

Characteristic	Included (n = 411)	Excluded (n = 234)	p-value
Age at IPT1 (years), mean (SD)	66.3 (8.5)	64.3 (8.3)	0.003
BMI (kg/m^2), mean (SD)	25.0 (3.3)	24.6 (3.5)	0.139
Female sex, n (%)	251 (61.1)	147 (62.8)	0.660
Ever smoker, n (%)	236 (58.3)	150 (64.9)	0.098
Stroke history, n (%)	6 (1.5)	2 (0.9)	0.511
Diabetes mellitus, n (%)	24 (6.0)	5 (2.2)	0.029
Serum biomarkers, mean (SD)			
Serum uric acid (mmol/L)	5.10 (1.39)	4.95 (1.25)	0.163
Serum albumin (g/dL)	4.54 (0.30)	4.51 (0.27)	0.253
Serum total protein (g/dL)	7.41 (0.47)	7.37 (0.42)	0.231

Excluded participants (n = 234): individuals with available IPT1 clinical examination records who were not included in the final analytic sample due to missing migraine-status and/or serum biomarker data.

[a] Ever smoker: any reported regular or occasional tobacco use.

Continuous variables compared using independent-samples Welch t-tests; categorical variables compared using Pearson chi-square tests. Bold p-values indicate $p < 0.05$.

Table S2. Sensitivity analyses: serum uric acid, albumin, and total protein by migraine status.

		Migraine (N=23), mean (SD)	No Migraine (N=388), mean (SD)	t	p-value
**Primary analysis (n = 411)**	Serum uric acid (mmol/L)	n = 23, 4.39 (0.86)	n = 378, 5.15 (1.41)	−3.93	<0.001
	Serum albumin (g/dL)	n = 23, 4.40 (0.26)	n = 370, 4.55 (0.30)	−2.64	0.008
	Serum total protein (g/dL)	n = 23, 7.16 (0.40)	n = 370, 7.43 (0.47)	−3.11	0.002
**Sensitivity 1: stroke excluded (n = 406)**	Serum uric acid (mmol/L)	n = 21, 4.39 (0.90)	n = 375, 5.15 (1.41)	−3.61	<0.001
	Serum albumin (g/dL)	n = 21, 4.37 (0.26)	n = 367, 4.55 (0.30)	−3.07	0.002
	Serum total protein (g/dL)	n = 21, 7.13 (0.39)	n = 367, 7.43 (0.47)	−3.40	<0.001
**Sensitivity 2: stroke, diabetes, gout, kidney disease, and liver disease excluded (n = 351)**	Serum uric acid (mmol/L)	n = 17, 4.42 (0.91)	n = 325, 5.09 (1.39)	−2.86	0.004
	Serum albumin (g/dL)	n = 17, 4.34 (0.26)	n = 318, 4.54 (0.29)	−3.15	0.002
	Serum total protein (g/dL)	n = 17, 7.11 (0.43)	n = 318, 7.44 (0.47)	−3.01	0.003

[a] Biomarker n differs from group n due to missing laboratory values.

Sensitivity 1: five participants with self-reported stroke history excluded.

Sensitivity 2: participants with self-reported stroke (n = 5), diabetes mellitus (n = 19), gout (n = 3), kidney disease (n = 32), or liver disease (n = 4) excluded; 60 participants removed in total (3 had overlapping conditions).

Abbreviations: SD, standard deviation. Independent-samples Welch t-tests used throughout. All results remained statistically significant across all sensitivity analyses.
